# Spatio-Temporal Dynamics of Viruses are Differentially Affected by Parasitoids Depending on the Mode of Transmission

**DOI:** 10.3390/v4113069

**Published:** 2012-11-12

**Authors:** Beatriz Dáder, Aránzazu Moreno, Elisa Viñuela, Alberto Fereres

**Affiliations:** 1 Associated Unit IVAS CSIC-UPM, Instituto de Ciencias Agrarias, CSIC, Serrano 115 Dpdo, 28006 Madrid, Spain; Email: beatrizdader@ica.csic.es (B.D.); amoreno@ica.csic.es (A.M.); 2 Associated Unit IVAS CSIC-UPM, Escuela Técnica Superior de Ingenieros Agrónomos, Universidad Politécnica de Madrid, Avda. Complutense s/n, 28040 Madrid, Spain; Email: elisa.vinuela@upm.es

**Keywords:** tritrophic interactions, biological control, *Aphis gossypii*, *Aphidius colemani*, CMV, CABYV, virus epidemiology, spatial dynamics, SADIE

## Abstract

Relationships between agents in multitrophic systems are complex and very specific. Insect-transmitted plant viruses are completely dependent on the behaviour and distribution patterns of their vectors. The presence of natural enemies may directly affect aphid behaviour and spread of plant viruses, as the escape response of aphids might cause a potential risk for virus dispersal. The spatio-temporal dynamics of *Cucumber mosaic virus* (CMV) and *Cucurbit aphid-borne yellows virus* (CABYV), transmitted by *Aphis gossypii* in a non-persistent and persistent manner, respectively, were evaluated at short and long term in the presence and absence of the aphid parasitoid, *Aphidius colemani*. SADIE methodology was used to study the distribution patterns of both the virus and its vector, and their degree of association. Results suggested that parasitoids promoted aphid dispersion at short term, which enhanced CMV spread, though consequences of parasitism suggest potential benefits for disease control at long term. Furthermore, *A. colemani* significantly limited the spread and incidence of the persistent virus CABYV at long term. The impact of aphid parasitoids on the dispersal of plant viruses with different transmission modes is discussed.

## 1. Introduction

Aphids (Hemiptera: Aphididae) are considered one of the most important pests worldwide not only because of the direct damage they cause, but also because their alimentary habits involve indirect damages. Most importantly, aphids are the primary vectors of plant viruses transmitting almost half of the known plant viruses, approximately 275 virus species within 19 different virus genera [1,2,3]. Transmission from plant to plant mediated by vectors is the most useful strategy for virus dispersal, as they are obligated parasites [3]. Plant viruses can be classified in two categories differing by the site in which the virus is retained by the vector and the retention period: non-circulative (with two different categories: non-persistent or stylet-borne viruses and semipersistent or foregut-borne viruses) and circulative or persistent viruses, which frequently accumulate in the salivary glands [1,4] .

Long distance movements of winged aphids could eventually lead to virus spread. Transient vectors that land and probe on a plant without colonising the crop are often the main vectors of non-persistent viruses, while colonising vectors are involved in transmission of persistent viruses [4]. Host preference first involves phototactic responses to visual cues that may be modified by the emission of plant volatiles [5,6]. The interaction between plant pathogens and vectors has been widely discussed but there is no general consensus on whether the parasite-induced changes in host phenotype favours vector’s responses such as settlement, behaviour, performance and overall fitness [6,7,8,9]. Non-circulative viruses exhibit different effects that mainly enhance vector attraction to infected hosts but some authors have documented neutral reproductive performance of vectors and their parasitoids reared on plants infected by circulative viruses [7,9].

Biological control (BC) is a major component of Integrated Pest Management (IPM) programs. Although natural enemies reduce the levels of herbivore pressure, their addition to a plant-virus-vector system may involve complicated interactions between the agents, as they may greatly modify disease incidence within the plant population [10]. Therefore, there is a need to balance BC consequences, as the benefits in reducing vector numbers may be offset by an increase in the spread of the virus. Several authors first studied the effect of natural enemies on virus spread by aphids throughout the plant population [11,12,13]. Roitberg and Myers [11] discussed the role of *Coccinella californica* Mannerheim (Coleoptera: Coccinellidae) in the spread of *Bean yellow mosaic virus* (*Potyviridae*, *Potyvirus*; BYMV). Bailey *et al*. [12] described how the predator activity of *Coleomegilla maculata* De Geer (Coleoptera: Coccinellidae) resulted in an increase in *Barley yellow dwarf virus* (*Luteoviridae*, *Luteovirus*; BYDV) incidence in oats. Similarly, Weber *et al*. [13] observed the increased ability of parasitised *Aphis fabae* Scopoli (Homoptera: Aphididae) to transmit *Beet yellows virus* (*Closteroviridae*, *Closterovirus*; BYV). This same trend has been confirmed in recent studies showing greater spread of *Pea enation mosaic virus* (symbiotic mutualism between an *Enamovirus* and an *Umbravirus*; PEMV) and BYMV by *Acyrthosiphon pisum* Harris (Homoptera: Aphididae) in the presence of the aphid parasitioid, *Aphidius ervi* Haliday (Hymenoptera: Braconidae) in *Vicia faba* L. [14,15].

Virus spread might be correlated with the foraging habit that natural enemies exhibit within the system [16,17]. Interestingly, the presence of the predator *Coccinella septempunctata* L. (Coleoptera: Coccinellidae) resulted in more BYDV-infected wheat seedlings, but conversely reduced virus incidence in the presence of the parasitoid *Aphidius rhopalosiphi* de Stefani Perez (Hymenoptera: Braconidae) probably because coccinellids have a more energetic searching behaviour [16]. Furthermore, certain species of aphids employ the ‘drop and move’ escape behaviour when they feel disturbed by foliar-foraging enemies [18,19], potentially increasing the risk of vector dispersal. Alarm pheromones play a crucial role in aphid dispersion and there have even been several attempts to mathematically model plant-virus-vector-natural enemy interactions by integrating this alarm signal, enhancing virus spread due to the presence of aphid parasitoids [20,21].

The distribution patterns of aphids and their natural enemies have highlighted the underlying dynamic relationships between guilds and their implications in biological control [22], as well as they have provided successful information about interplant movement of different aphid morphs [23]. The Spatial Analysis by Distance IndicEs (SADIE) methodology has proved itself to be one of the most powerful tools for studying distribution patterns [24,25,26,27]. The basis of SADIE is to measure the minimum value in terms of distance traveled, which individuals would have to move so that all they are spaced as uniformly as possible, known as *D* or distance to regularity. Previous studies of the spatial spread of major viral diseases affecting valuable outdoors crops have been studied using the SADIE methodology [28,29].

The present study aimed to investigate the tritrophic interactions within a system that included the host plant *Cucumis sativus* L., the cotton aphid *Aphis gossypii* Glover, a cosmopolitan pest species that colonises more than 600 host plants [30], and the widely used parasitoid wasp *Aphidius colemani* Viereck (Hymenoptera: Braconidae) [31]. Furthermore, parasitoid-mediated effects on the dissemination of two major plant viruses infecting Cucurbitaceae [32,33], *Cucumber mosaic virus *(*Bromoviridae*, *Cucumovirus*; CMV) and *Cucumber aphid-borne yellows virus* (*Luteoviridae*, *Luteovirus*; CABYV), both efficiently transmitted by *A. gossypii* in non-circulative [34] and circulative manner [35], respectively, were assessed. Additionally, the spatial distribution of both viruses and the degree of association between the two viruses and its vector was analysed under two different time frame scenarios

## 2. Results and Discussion

### 2.1. Effect of *Aphidius Colemani* on Aphid Dispersal and the Spread of Cucumber Mosaic Virus

**Figure 1:**
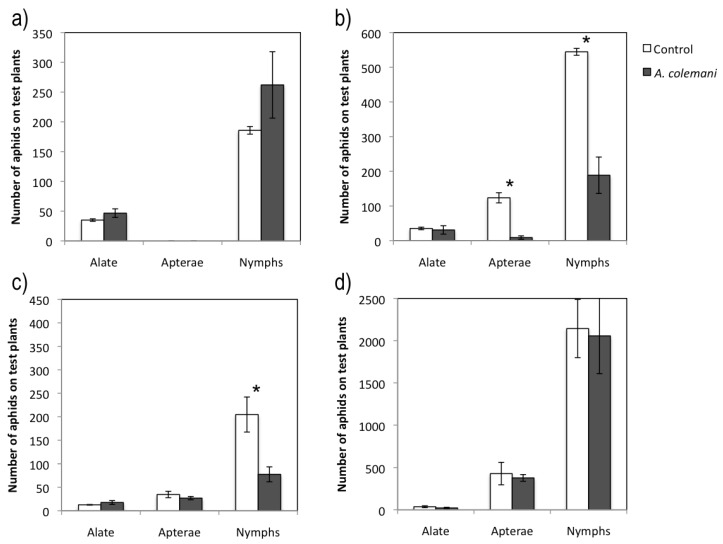
Mean ± S.E. values of total number of aphids on test plants in cages with (grey bars) and without (control cages, white bars) *A. colemani*. (**a**) CMV-infected source plant assay at 2 days. (**b**) CMV-infected source plant assay at 7 days. (**c**) CABYV-infected source plant assay at 7 days. (**d**) CABYV-infected source plant assay at 14 days. Bars with asterisks are significantly different according to Student’s *t* test (*p *< 0.05).

The population density of adult morphs and nymphs in the CMV-infected source plant located in the middle of the cage where aphids were released was frequently higher in the control cages than in those containing the parasitoid *A. colemani*, although no significant differences were found (Table 1). Parasitoids successfully located a variable number of aphids in the virus-infected source plant and mummies could be observed (2.3 ± 1.5) 7 days after the release of parasitoids, whereas they could not be detected after 2 days, as mummies were not yet developed. There were fewer aphids on the peripheral test plants in the arenas (= cages) without *A. colemani* after 2 days (Figure 1a), but this trend was not repeated after 7 days, with significantly more apterae adults (*t *= 6.775; 4 gl; *p *= 0.002) and nymphs (*t *= 11.864; 4 gl; *p *< 0.001) in the test plants of control arenas (Figure 1b). The number of nymphs increased considerably after 7 days (Figure 1b). Besides, recognizable mummies could be detected in peripheral plants in CMV at 7 days (2.0 ± 2.0) but not at 2 days.

**Table 1:** Mean ± S.E. density (number of individuals/plant) of adult morphs and nymphs in the CMV-infected virus source plant after 2 and 7 days in cages with and without (control) *A. colemani* followed by statistics according to Student’s *t* test (*p *< 0.05).

	2 days				7 days			
	Control	*A. colemani*	*t*	*p*	Control	*A. colemani*	*t*	*p*
Alate	24.7 ± 5.5	18.0 ± 5.3	0.873	0.947	11.0 ± 3.2	9.0 ± 2.5	0.524	0.628
Apterae	-	-	-	-	10.7 ± 4.7	6.3 ± 3.0	0.882	0.428
Nymphs	176.3 ± 55.1	128.7 ± 26.6	0.745	0.498	170.0 ± 50.0	99.7 ± 33.3	1.227	0.287

The occupancy rates were calculated as the percentage of test plants with one or more aphids in their different morphs. These rates were consistent with aphid density in the peripheral test plants (Figure 1a and 1b), as there were significantly fewer plants occupied by aphids in the control arenas than in the arenas with parasitoids after 2 days, but larger occupancy rate in the control after 7 days (Table 2).

**Figure 2:**
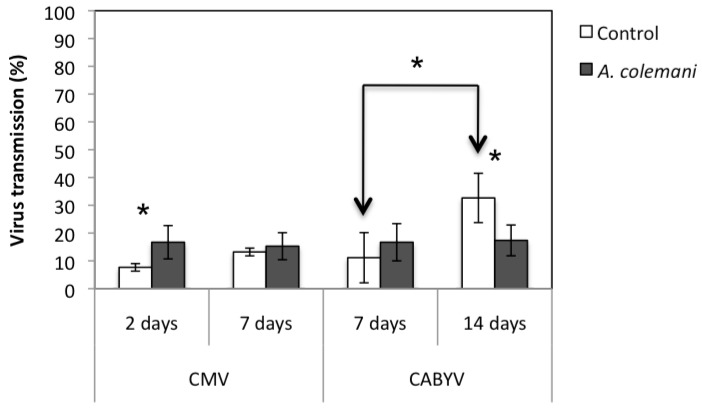
Mean ± S.E. values of virus transmission (%) in the arenas with parasitoids (grey bars) and in those without them (control, white bars). Bars with asterisks indicate significant differences according to a Chi-square 2 × 2 goodness of fit test (*p *< 0.05).

The incidence of CMV at short time (after 2 days) was significantly higher in arenas containing *A. colemani* compared to that observed in the control arenas (*X^2 ^*= 5.497; *p *= 0.020). When the same assay was assessed after 7 days, no significant differences in the incidence of CMV between treatments were detected (Figure 2).

**Table 2:** Mean ± S.E. percentage of test plants occupied by one or more alate, apterae or nymphs (occupancy rate) in CMV-infected source plant assays after 2 and 7 days in cages with and without (control) *A. colemani*. Means in the same row followed by statistics in bold are significantly different according to a Chi-square 2 × 2 goodness of fit test (*p *< 0.05).

	2 days				7 days			
	Control	*A. colemani*	*X^ 2^*	*p*	Control	*A. colemani*	*X^ 2^*	*p*
Alate	38.9 ± 2.5	54.2 ± 7.9	**9.930**	**0.002**	42.4 ± 3.5	33.3 ± 8.7	2.495	0.117
Apterae	-	-	-	-	38.2 ± 2.8	6.3 ± 3.2	**42.509**	**<0.001**
Nymphs	38.2 ± 4.2	50.7 ± 8.2	**4.556**	**0.044**	50.7 ± 2.5	38.2 ± 7.8	**4.556**	**0.034**

Overall, our results suggest that *A. colemani* promoted early movement of *A. gossypii* from the virus-infected source towards the peripheral plants. Parasitoids significantly increased the colonization of adjacent plants by both adults and nymphs. Consistently, the spread of CMV also increased when parasitoids interacted with aphids for 2 days. However, no differences in virus incidence could be found after 7 days between both treatments and aphids spread around and transmitted CMV equally well under the two treatments (in the presence and absence of *A. colemani*). These results may be explained by the mode of transmission of non-persistent viruses and the particular behaviour of *A. gossypii*. Transmission of non-persistent viruses is highly favoured if aphids acquire the viral particles during short periods, decreasing its efficiency with a longer acquisition access time [4]. It is likely that soon after release, parasitoids caused a disturbance and their presence forced aphids to quickly disseminate, which led to an increase in aphid density on peripheral test plants and subsequent transmission of CMV. *A. colemani* forced them to escape and, as non-persistent viruses do not require a latent period, aphids were able to inoculate CMV to peripheral plants readily after leaving the central plant. The incidence of CMV remained the same 2 and 7 days after the experiment started in arenas with *A. colemani*, probably because parasitised aphids had reduced the distance they moved after few days and parasitoids were not in the attack mood any longer. The fact that mummies were observed in both central and test plants at 7 days seems to corroborate this hypothesis. Conversely, CMV transmission in control arenas increased with time as aphids continued colonizing test plants because their mobility was not jeopardised by parasitism. Similar alterations resulting in an increase in non-persistently and semipersistenly transmitted virus dispersal have been previously reported at short times (1–3 days) [11,13,14,17], but no information on the long term effects of parasitism on virus spread has been reported so far.

Natural enemies orientate towards plant-host complexes (PHC) by responding to host herbivore-induced plant volatiles and visual cues [36,37,38,39]. The emission of alarm signals by aphids causes conspecifics to disperse through different strategies such as ‘drop off’ [18,19]. That escaping behaviour may even modify virus spread and has been reported with the aphid *A. pisum* and its parasitoid *A. ervi* [20]. Furthermore, damaged plants emit the “cry-for-help” signal that may indirectly benefit hosts under herbivore attack [21]. Therefore, it is evident that there is a need to integrate all these multitrophic reactions between agents resulting in epidemiological consequences [20,21].

**Figure 3:**
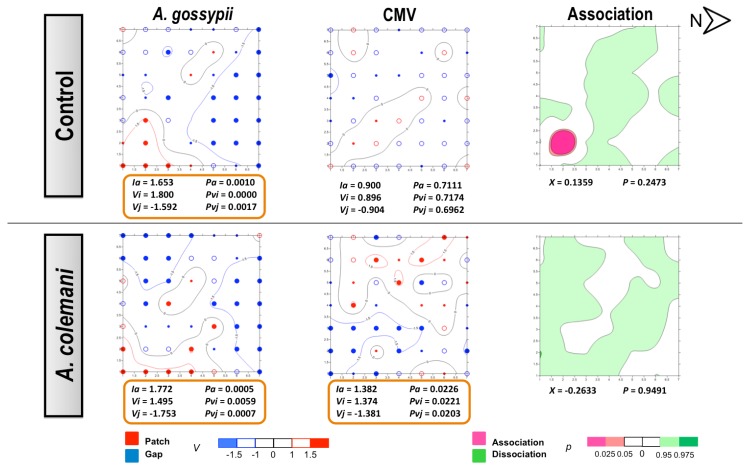
Classed post maps of the spatial distribution of mean number of *A. gossypii* and cumulative number of CMV-infected plants (total number of infected plants per treatment) at 2 days, and contoured map of the association between CMV-infected plants and its vector, *A. gossypii*. Spots indicate individual test plants. Small filled spots represent clustering indices of 0 to ±0.99 (clustering below expectation), unfilled spots ±1 to ±1.49 (clustering slightly exceeds expectation) and large filled spots >1.5 or <1.5 (more than half as much as expectation). Red lines enclosing patch clusters are contours of *v *= 1.5 and blue lines are of *v *= –1.5. Black lines are zero-value contours, representing boundaries between patch and gap regions. The index of aggregation, *Ia*, the positive patch cluster index, *vi*, the negative gap cluster index, *vj*, and the index of spatial association, *X*, enclosed by an orange line are statistically significant. Letter N and arrow indicate north orientation.

**Figure 4:**
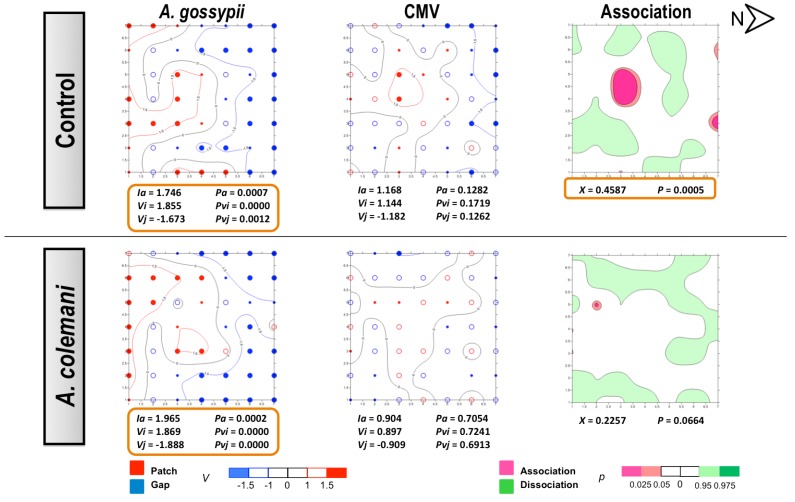
Classed post maps of the spatial distribution of mean number of *A. gossypii* and cumulative number of CMV-infected plants (total number of infected plants per treatment) at 7 days, and contoured map of the association between CMV-infected plants and its vector, *A. gossypii*. Symbols and contours are as for Figure 3.

No differences could be found for the mean displacement (δ) of both aphids and CMV (Table 3). The spatial analysis of CMV-infected source plant experiments showed a significant aggregated distribution of *A. gossypii* in treatments with and whitout parasitoids after 2 and 7 days (Figure 3 and Figure 4). At 2 days, aphids were restricted to the lower right corner of the experimental arena (Figure 3) but colonised the entire lower area of the arena after 7 days, with plants remaining unnoccupied in the upper side (Figure 4). At short term, the spread of CMV followed a regular distribution in the control cages where few isolated plants became infected, although CMV distribution was significantly aggregated in the presence of parasitoids (Figure 3). When the spatial distribution of aphids and CMV was studied at long term (7 days), opposite results were obtained (Figure 4). The combination of aphid infestation and virus infection showed a significant association in the control arenas, that was statistically significant at 7 days (Figure 4), and a significant dissociation in the the presence of parasitoids after 2 days (Figure 3).

**Table 3:** Mean ± S.E. values of the displacement (δ) of aphids and CMV after 2 and 7 days in arenas with and without (control) parasitoids, followed by statistics according to Student’s *t* test (*p *< 0.05).

	Aphids				Virus			
	Control	*A. colemani*	*t*	*p*	Control	*A. colemani*	*t*	*p*
2 days	1.9 ± 0.2	1.6 ± 0.2	0.758	0.491	0.9 ± 0.1	1.4 ± 0.6	–0.690	0.528
7 days	1.3 ± 0.1	1.5 ± 0.4	–0.539	0.619	1.2 ± 0.4	1.0 ± 0.4	0.215	0.840

In our experiments, *A. gossypii* showed the typical pattern of a colonizing aphid vector species because of its strong preference of cucumber as a host [30]. However, the spread and incidence of the virus progressed differently at short and long times of evaluation. The contoured maps of CMV after 2 days revealed the typical pattern of a non-persistent virus in the absence of parasitoids (control cages). However, the spatial distribution was modified in the presence of *A. colemani* and the clear consequence of the immediate disturbance of aphids, which promoted the distribution of CMV around the entire arena and its aggregation in several patches, in contrast with the few red spots indicating isolated infections under the control arenas. At 7 days, the infection in control arenas showed how the initial localised foci had merged in a larger patch, whereas a regular distribution (*Ia *< 1) was found in the presence of parasitoids. The *A. gossypii*-infested and CMV-infected plants were significantly associated in the control arenas, whilst *A. colemani* induced dissociation between both agents, highlighting again the strong effect of natural enemies in the early dispersal of aphids as previously reported [11,14].

### 2.2. Effect of *Aphidius Colemani* on Aphid Dispersal and the Spread of Cucumber Aphid-Borne Yellows Virus

The short and long term experiments with CABYV was extended to 7 and 14 days, respectively, because its circulative mode of transmission requires longer acquisition and inoculation access periods than the non-circulative CMV. The central CABYV-infected plant contained more aphids in the control arenas than in those with *A. colemani*, and significant differences in the number of apterae adults and nymphs were detected at 7 days, but not at 14 days (Table 4). Moreover, it was possible to detect mummies in the virus source plant 14 days (8.3 ± 2.0) but not 7 days after parasitoid release. In general terms, there were more peripheral test plants occupied by a greater number of aphids in the control arenas than in those containing *A. colemani*, with significant differences in the case of nymphs at 7 days (*t *= 3.152; 4gl; *p *= 0.034) (Figure 1c). However these significant differences were not observed at 14 days (Table 5, Figure 1.d). As mentioned before for the CMV experiments, the number of offspring was much higher at long term (Figure 1.d). Mummies (56.0 ± 21.6) and even parasitoid adults (3.3 ± 1.8) were found in peripheral test plants at 14 days again but not at 7 days. The mean incidence of CABYV when evaluated at short time (7 days) was higher in arenas containing *A. colemani* compared to that observed in the control ones, although no significant differences were found. At long term, significantly fewer CABYV-infected plants were detected in arenas where *A. colemani* were introduced (*X^2 ^*= 8.963; *p *= 0.004) (Figure 2). 

The overall mean transmission rate of both viruses (CMV and CABYV) in the presence and absence of *A. colemani* was not significantly different after 7 days. However, the presence of *A. colemani* increased the rate the spread of CMV at short term (2 days) but reduced the spread of CABYV at long term (14 days) (Figure 2). Moreover, the transmission rate of CABYV in control cages significantly increased at 14 days compared to 7 days from 11.1 ± 9.0% to 32.6 ± 8.9% (*X^2 ^*= 19.525; *p *< 0.001) (Figure 2).

**Table 4:** Mean ± S.E. density (no. of individuals/plant) of adult morphs and nymphs in the CABYV-infected source plant at 7 and 14 days in cages with and without (control) *A. colemani*. Means in the same row followed by statistics in bold are significantly different according to Student’s *t* test (*p *< 0.05).

	7 days				14 days			
	Control	*A. colemani*	*t*	*p*	Control	*A. colemani*	*t*	*p*
Alate	36.0 ± 1.7	24.3 ± 7.8	1.443	0.222	20.0 ± 6.7	10.0 ± 1.2	1.359	0.246
Apterae	60.3 ± 9.9	17.0 ± 1.5	**6.524**	**0.003**	179.7 ± 54.3	185.0 ± 8.2	0.168	0.874
Nymphs	388.0 ± 39.1	160.0 ± 10.4	**7.026**	**0.020**	1624.7 ± 297.8	1212.0 ± 55.6	1.465	0.217

**Table 5:** Mean±S.E. percentage of test plants occupied by one or more alate, apterae or nymphs (occupancy rate) in CABYV assays at 7 and 14 days in cages with and without (control) *A. colemani* followed by statistics according to a Chi-square 2x2 table test goodness of fit test (*p* <0.05).

	7 days				14 days			
	Control	*A. colemani*	*X^ 2^*	*p*	Control	*A. colemani*	*X^ 2^*	*p*
Alate	18.8 ± 1.2	24.3 ± 4.2	1.315	0.256	37.5 ± 9.8	30.6 ± 9.1	1.547	0.263
Apterae	20.9 ± 5.2	16.0 ± 3.0	1.133	0.293	63.9 ± 15.2	60.4 ± 1.2	0.369	0.627
Nymphs	27.8 ± 2.8	29.8 ± 4.6	0.152	0.670	75.0 ± 11.0	72.2 ± 0.7	0.286	0.596

Previous studies have described enhanced spread of persistent viruses in the presence of natural enemies [12,15], although the vector response might be influenced by the natural enemy’s searching habits [16]. At 7 days, there were no differences in virus incidence between the two treatments, however significantly fewer CABYV-infected plants were observed at 14 days in the presence of *A. colemani*, the same as seen by Smyrnioudis *et al*. [16] for both predators and parasitoids. Despite the fact that some studies show that virus transmission rate is not reduced by parasitoids [40], Calvo and Fereres [9] reported a reduction in the rate of spread of a circulative *Polerovirus* due to a decrease in the life span of viruliferous aphids in the presence of parasitoids. In our study, we found, that mummification might have positively diminished the duration of aphids as active vectors after 14 days, so CABYV incidence was significantly reduced in comparison to that in the control cages. The recording of mummies and parasitoid adults provided evidence that *A. colemani* was able to establish itself well in the experimental arenas. It has been reported an average of 10 days at a constant temperature of 25 °C for *A. colemani* to complete its development [41]. With the mean temperatures in which our experiments were performed, a new generation of parasitoids could have started to emerge after 14 days so that the reduction in vector populations may have possibly limited further virus spread.

**Figure 5:**
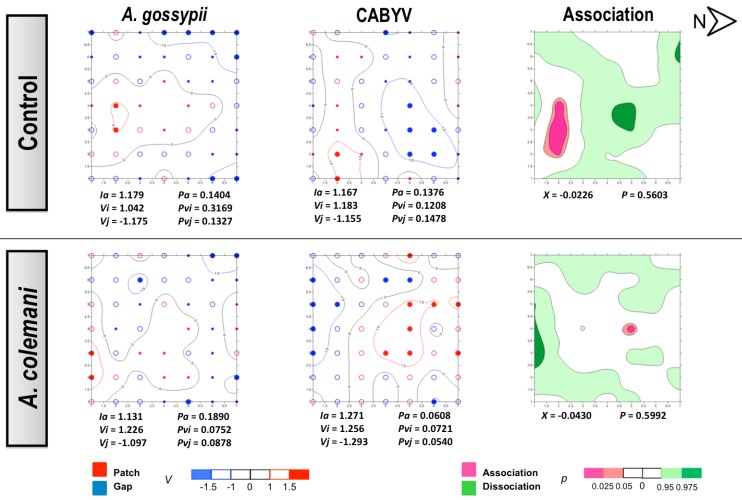
Classed post maps of the spatial distribution of mean number of *A. gossypii* and cumulative number of CABYV-infected plants (total number of infected plants per treatment) at 7 days, and contoured map of the association between CABYV-infected plants and its vector, *A. gossypii*. Symbols and contours are as for Figure 3.

**Figure 6:**
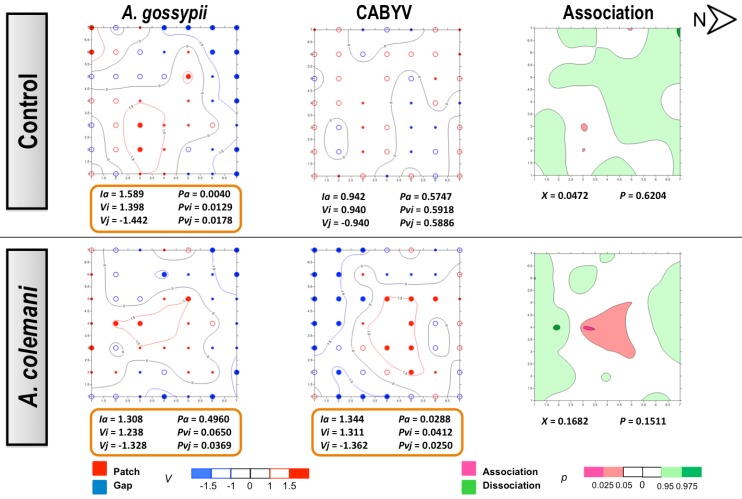
Classed post maps of the spatial distribution of mean number of *A. gossypii* and cumulative number of CABYV-infected plants (total number of infected plants per treatment) at 14 days, and contoured map of the association between CABYV-infected plants and its vector, *A. gossypii*. Symbols and contours are as for Figure 3.

The same as in CMV experiments, no differences could be found for the mean displacement δ of both aphids and CABYV-infected plants under the two treatments (Table 6). Aphids also showed an aggregated distribution in the CABYV assays, being significantly aggregated at 14 days (Figure 6) as cucumber is an excellent host plant for *A. gossypii* and large colonies are produced in a short period of time [30]. Aphid spatial distribution showed a very similar pattern in the CABYV experiments when parasitoids were present, with population moving to the southern area of the cages and increasing the number of occupied plants occupied as time progressed (Figure 5 and Figure 6). In the control arenas, aphid distribution was limited to the south and center of the experimental cage at short times (Figure 5) but reached almost all edges when aphids were allowed to stay 14 days, in parallel to what happened with the spread of CABYV (Figure 6). Conversely, virus-infected plants were located in the northern area in arenas with parasitoids at 7 days (Figure 5), and continued being restricted in the same area with a significantly aggregated distribution at 14 days (Figure 6). When the spatial distribution of the virus and the vector were combined, a dissociation at 7 days and association at 14 days between aphid location and the position of CABYV-infected plants were recorded in both treatments (Figure 5 and Figure 6).

**Table 6:** Mean ± S.E. values of the displacement (δ) of aphids and CABYV after 7 and 14 days in arenas with and without (control) *A. colemani*, followed by statistics according to Student’s *t* test (*p *< 0.05).

	Aphids				Virus			
	Control	*A. colemani*	*t*	*p*	Control	*A. colemani*	*t*	*p*
7 days	0.8 ± 0.1	1.2 ± 0.2	–1.496	0.209	2.2 ± 1.0	1.0 ± 0.3	1.154	0.313
14 days	0.8 ± 0.3	0.8 ± 0.1	–0.112	0.916	1.4 ± 0.4	1.0 ± 0.0	1.117	0.326

In the CABYV assays, there were major clustered areas of either patches or gaps of infected plants at 7 days in both treatments. Patch clusters seemed to be wider than gap ones in the *A. colemani* treatment. Clusters of infected plants are frequently observed in viruses transmitted in a persistent circulative manner [42]. Moreover, just two weeks were enough for aphids to expand CABYV to all the edges of the experimental arena in the absence of *A. colemani,* whilst parasitoids limited the incidence of CABYV to specific patches. These results, together with the reduction in the rate of transmission of CABYV in the presence of *A. colemani* after 14 days, prove the beneficial role of natural enemies at long term, specially when dealing with viruses transmitted in a persistent circulative manner.

The evaluation periods were selected depending on the type of transmission of each virus-vector combination and the life history of *A. colemani*. These periods cannot be comparable to the crop’s growth cycle that approximately lasts three to four months in commercial greenhouses. Making a prediction in a hypothetical scenario where the experiments had be run for a longer period is adventurous, but all plants would have possibly become infected by the persistent virus CABYV if parasitoids had not been released in the arena. Our data suggests that the spread of CABYV would be strongly constrained in the presence of parasitoids under a real field situation at long term because the mobility and population growth of aphid vectors would be jeopardised. However, it would be more difficult to predict the long-term consequences of parasitoid release in the case of the non-persistent virus CMV. Further research would be helpful to assess the effect of parasitism on aphid dispersal and virus transmission over time.

This study refers to a specific virus-vector-host-natural enemy complex, but natural enemy diversity occurring in cucurbit crops could influence the spread of both viruses in either direction. As a first approach, it will be desirable to carry out further studies with different natural enemies to study how this beneficial guild could modify viral dynamics and to investigate differences between their behaviour. As a second step, different virus-vector combinations could be evaluated to gain a better understanding of multitrophic interactions in pathosystems where whiteflies or thrips are present.

## 3. Experimental Section

### 3.1. Insect Cultures

A single virginiparous aptarae collected in a melon crop in Almería (Spain) in 1998 was used to initiate a clonal population of *Aphis gossypii*. The aphid colony was maintained at ICA-CSIC (Madrid, Spain) (Lat. 40°43′97″N, Long. 3°68′69″W, Alt. 710 m) on *Cucumis melo* L. cv. Primal plants at low density in a climate chamber at 23:18 °C temperature (day:night), 60–80% RH, and a photoperiod of 16:8 (light:dark). Alate aphids from the colony were collected with an aspirator and grouped in falcon tubes the same morning where the experiment was conducted.

*Aphidius colemani* adults, kindly supplied by Koppert España SL (Almeria, Spain), were cultured in climatic chambers at ICA-CSIC using *A. gossypii* colonies on *Cucumis sativus* cv. Ashley according to the methodology described by Calvo and Fereres [9]. Young *A. colemani* females exhibiting oviposition attack behaviour when introduced in a plastic tube with a leaf infested by *A. gossypii* were selected and placed in falcon tubes the same day where the experiment was conducted.

### 3.2. Virus Sources and Test Plants

Cucumber, *C. sativus* cv. Marumba plants were inoculated with CMV and CABYV 13 days after sowing at the 1-true leaf stage and used 4 weeks post-inoculation as viral sources. Plants were mechanically inoculated with CMV isolate M96/9 (IA subgroup), obtained from a melon crop in 1996 in Tarragona (Spain) and kindly provided by Dr. E. Moriones (EELM-CSIC, Spain) [43]. CMV-infected cucumber plants were maintained at ICA-CSIC in an insect-proof chamber at 25:20 °C (day:night), a photoperiod of 16:8 (light:dark) and 90% HR. The CABYV isolate kindly provided by H. Lecoq, was obtained in Montfavet, France, from zucchini squash in 2003, and maintained in cucumber plants at ICA-CSIC by aphid serial transmission. In summary, *A. gossypii* adults were allowed to feed for 2 days on previously CABYV-infected plants and then were transferred to healthy plants as described in Moreno *et al*. [44]. Then, the adult aphids were removed from the infected source plants after 48 hours and nymphs produced during this period were allowed to feed on the CABYV-infected plants for one extra day, reaching an acquisition access period (AAP) of three days. After the AAP, 25 nymphs grown on the CABYV-infected plants were transferred to each healthy receptor plant for an inoculation access period (IAP) of 3 days. After the IAP, plants were sprayed with Biopirot® (Aragonesas Agro). CABYV-inoculated plants were grown in greenhouse facilities at 20:16 °C (day:night), a photoperiod of 16:8 (light:dark) and 60% HR until experiments took place.

Infection was confirmed by Double Antibody Sandwich Enzyme-Linked ImmunoSorbent Assay (DAS-ELISA) [45] using specific commercial antibodies against CMV (Agdia Inc., Indiana, USA) and CABYV (Sediag, France). 

Cucumber test plants were germinated in 10.5 cm diameter pots filled with a 50:50 mixture of vermiculite (No. 3, Asfaltex S.A., Barcelona, Spain) and soil substrate (Kekilla Iberica, Almería, Spain). Plants were watered three times a week using 20-20-20 (N:P:K) Nutrichem 60 fertilizer (Miller Chemical & Fertilizer Corp., Pennsylvania, USA) at a 0.25 g/L dosage. Plants were grown in an insect-proof chamber at 25:20 °C temperature (day:night), a photoperiod of 16:8 (light:dark) and 60–80% HR. 

### 3.3. Effect of *Aphidius Colemani* on Aphid Dispersal and Virus Spread

**Figure 7:**
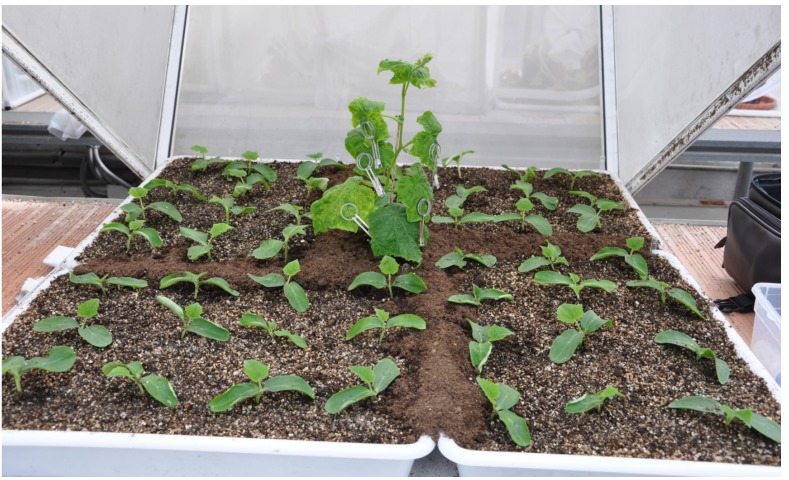
Spatial disposition of an arena, displaying the central virus source surrounded by the 48 test plants at a 1-true leaf stage.

Four different assays were carried out in greenhouse facilities at the ICA-CSIC to evaluate the impact of the parasitoid *A. colemani* on the spread of CMV and CABYV and its vector *A. gossypii* at short and long term. Temperature, relative humidity and shadow covers were remotely controlled in the greenhouse through a central computer that ensured the following mean environmental conditions: 21–23 °C temperature, a photoperiod of 16:8 (light:dark) and 75–80% HR. A set of 6 cages (1 × 1 × 1 m) (3 replicates, 2 treatments) as described by Díaz *et al*. [23] was used in each experiment. A potted CMV- or CABYV-infected plant was placed in the centre of each cage (Figure 7). Four trays with holes on the bottom to allow percolation containing 48 test plants with a planting distance of 12.5 cm within and between rows were placed surrounding the virus source. The surface was covered with a uniform layer of soil to create a continuous surface.

One hundred winged aphids were released in each virus infected source-plant using clip-cages to allow an acquisition access period of 15 minutes. After this period, clip-cages were removed and a falcon tube with five female parasitoids was introduced in the experimental arena. A vial with diluted honey (1:1 by volume) was placed inside the cages as a feeding source for parasitoids. Control cages were similarly implemented with infected-sources and test plants without the introduction of parasitoids. All cages were rotated 180° daily to allow aphid distribution as uniform as possible and avoid orientation bias.

Number, status and position of aphids were recorded in test plants at different periods of time (short and long term) depending on both the mode of transmission of the virus studied and the life history of the parasitoid [41]. As a first approach, assays with a CMV and CABYV virus source were evaluated after 2 and 7 days (short term), respectively. CMV is acquired and transmitted during brief periods of time and without a latent period so both processes could occur in that short time periods (few days). Conversely, acquisition and transmission of circulative viruses require vectors to feed during a much longer period of time. Moreover, circulative viruses exhibit a delay in hours to days between the moment in which the vector feeds on the infected plant and their ability to transmit the virus, which ranges from several days to weeks. In the second set of bioassays, the virus-vector-parasitoid complex was allowed to evolve 7 days in the case of CMV and 14 days for CABYV to evaluate long-term virus and vector dispersion. Additionally, the number and position of mummies and parasitoid adults were recorded in the long-term assays as a new cycle of parasitism was established and mummies were easily recognizable. The virus source plant was removed after each specific time period and the number, status and position of aphids was recorded. Then, all test plants were sprayed with Confidor® 20 LS (Bayer CropScience) to avoid further virus transmission. Four weeks after the experiment was completed, virus infection in each of the receptor plants was tested by DAS-ELISA as described above.

### 3.4. Statistical Methods and Spatial Analysis

Density, stage and morph of aphids (winged or wingless adults or nymphs) in both central and receptor test plants were analysed using the IBM Statistics SPSS software [46]. Data was previously transformed with either √(x + 0.5), x^2^ or Ln (x + 1) in order to decrease heteroscedasticity and achieve normal distribution. All the parameters were then compared between the control cages and those containing parasitoids through a two sample Student’s *t* test for comparisons of means (*p *≤ 0.05). The proportion of plants infested by 1 or more aphids (occupancy rate) and virus incidence in test plants were compared between the two treatments (with and without parasitoids) by using a Chi-square 2 × 2 table test goodness of fit test (*p *≤ 0.05) to check if the observed frequency distribution was related to the expected frequency distribution.

Spatial distribution of aphids and virus spread was studied using the SADIE methodology, which was introduced to replace the traditional mathematical approach by a more understandable biology-based measure [24]. SADIE calculates the distance to regularity, *D*, which is the minimum value of the total distance that individuals have to move, from unit to unit, so that the population are dispersed as regularly as possible. Also the method provides the distance to crowding, *C*, as the minimum value of the total distance that individuals must move to be as aggregated as possible [25]. 

The spatial pattern of data was described by the index of aggregation, *Ia*, the positive patch cluster index, *vi*, and the negative gap cluster index, *vj* [27]. *Ia* denotes the pattern of the population which by convention is an aggregated sample if *Ia *> 1, a spatially-random sample if *Ia *= 1, and a regular sample if *Ia *< 1. For units within patches of relatively large counts close to another, the patch cluster index would be large. Conversely, the gap cluster index tends to be large in units within gaps of small counts close to another. Clustering indices may be plotted on a map as they are correlated on a continuous scale. Both indices visually indicate the location and extent of cluster in the data so their values could be contoured with Surfer 9.0 [47] that allows the graphical representation of patches and gaps. Moreover, the degree of local association between aphid population and virus incidence was calculated with the index of spatial association, *X*, and contoured as well [26]. In our study, the assessment of aphid density was determined by the average of the three cages of each treatment in every position. Virus incidence was represented as the cumulative number of infected plants in the three cages under each treatment in every position. A value of 1 and 0 was assigned to the infected plants and healthy plants, respectively. Centroids, *C*, or the average position of either aphids or viruses in the experimental cage, were used to calculate δ, the displacement of the entire populations between centroids and the place where both aphids and virus-infected plants were originally located (the central virus source).

## 4. Conclusions

The parasitoid *A. colemani* promoted early dispersal of aphids and subsequent increase in the incidence of the non-persistent virus CMV at short term (2 days), though consequences of parasitism suggest potential benefits at a long term. Furthermore, introduction of *A. colemani* significantly limited the spread of the circulative virus CABYV, at long term (14 days). In cages with parasitoids, CABYV had a restricted distribution whereas infection spread all over the experimental arena in the control cages, proving the beneficial role of parasitoids in limiting the spread of both aphids and associated viruses transmitted in a persistent manner. Our study shows that the reduction of herbivore damage at long term may offset the initial risk of potential virus dispersal when natural enemies first encounter their hosts/preys.

It is clear that the outcome in our system might be also influenced by the vector preference for healthy or diseased plants, the plant response to pest damage caused by vectors in attracting parasitoids (cry-for-help signal) and the vector response to the presence of parasitoids (alarm signal) at short as well as at long term [20]. The infected-host attractiveness mediating settlement and arrestment behaviours constitute a key point in this process because both of them are correlated to the time required to positively acquire the viral particles [6,7,8]. It is know that both persistent (e.g. CABYV) and non-persistent (e.g. CMV) viruses would tend to enhance vector attraction to infected plants (increasing alighting and arrestment of their vectors). However, it is known that both type of viruses have contrasting effects on vector settling and feeding preferences, with persistent viruses tending to improve host quality for vectors and promote long-term feeding while non-persistent viruses tend to reduce plant quality and promote rapid disersal [48]. Moreover, in our experiments aphids released on the virus-infected source plant were non-viruliferous. It has been shown that aphids infected with another circulative *Luteovirus* (BYDV) prefer to settle on virus-infected than on non-infected plants [49]. All together it seems that both non-persistent and persistent viruses have coevolved and adapted to exploit specific behavioral traits of their vectors to enhance their own spread. Thus the spatial and temporal pattern of virus spread will depend on the mode of transmission (persistent or non-persistent) as well as on the “infection” status of the virus vector.

Overall, these observations suggest the importance of taking into account the degree of activity of natural enemies when implementing IPM programs for controlling vectors of plant diseases.
